# Chinese version of exercise dependence scale-revised: psychometric analysis and exploration of risk factors

**DOI:** 10.3389/fpsyg.2023.1309205

**Published:** 2023-12-05

**Authors:** Yingbo Shao, Haoyu Zhang, Xiaonan Zhang, Qian Liang, Hui Zhang, Feifei Zhang

**Affiliations:** ^1^Department of Radiology, First Hospital of Shanxi Medical University, Taiyuan, Shanxi, China; ^2^Department of Medical Imaging, Shanxi Medical University, Taiyuan, Shanxi, China; ^3^Shanxi Key Laboratory of Intelligent Imaging and Nanomedicine, First Hospital of Shanxi Medical University, Taiyuan, Shanxi, China

**Keywords:** exercise addiction, exercise motivation, psychometric, reliability, validity

## Abstract

**Introduction:**

Exercise addiction (EA) is a dysfunctional behavior characterized by exaggerated training which has adverse effects on physiology and psychology. To examine the reliability and validity of the Chinese version of the Exercise Dependence Scale-Revised (EDS-R) and the social and psychological aspects related to EA, a large sample behavioral study was conducted.

**Methods:**

College students were selected as the target group. All of them were asked to finish the scales about exercise, including the Chinese version of EDS-R and the Motives for Physical Activity Measure-Revised scales. A confirmatory factor analysis, Mann-Whitney U test, and hierarchical regression test were performed to test the reliability and validity of the Chinese version of EDS-R and find the explanatory variables of EA.

**Results:**

A total of 837 (556 female) students with a mean age of 20.38 years were recruited in the present study. The Chinese version of EDS-R showed good reliability and validity (McDonald’s *ω* = 0.973, CR = 0.99, AVE = 0.80) in Chinese college students. EA was positively correlated with exercise frequency and ability motivation across the study sample. Besides, the Mann-Whitney U test revealed that the exercise motivation difference is the primary cause of the gender gap in EA.

**Conclusion:**

The Chinese version of EDS-R is a relatively robust and accurate instrument to assess the risk of EA. Additionally, exercise frequency and motivation may be the potential risk factors for EA. The screening of risk factors is of great significance for the early detection and prevention of EA.

## 1 Introduction

It is widely recognized that regular physical activity is an effective means of promoting both physical and mental health (Landolfi, 2013; Lynch et al., 2013; Hausenblas et al., 2017). The current physical activity guidelines formulated by the World Health Organization (WHO) and the American College of Sports Medicine (ACSM), establish the minimum amount of physical activity necessary to achieve health benefits. Recommend that adults engage in at least 150 min of moderate-intensity aerobic physical activity or 75 min of vigorous-intensity aerobic physical activity per week (Piercy et al., 2018). The guidelines emphasize that further physical activity beyond the minimum recommendations may provide additional health benefits, but they do not provide specific upper limits. The benefits of exercise are proportional to an increase in the amount of exercise, but this collinearity stops when the negative consequences of excessive exercise appear (Costa et al., 2012). In some cases, exercise may be used as a compensatory behavior for regulating emotions or controlling body weight, or it may occur independently of other disorders and be driven by the pleasurable effects of physical activity, leading to exercise addiction (EA) (Trott et al., 2022). According to the American Psychological Association Diagnostic and Statistical Manual of Mental Disorders, 4th Edition (DSM-4) (Bell, 1994), EA is characterized by an inability to satisfy a persistent and escalating desire for physical activity, despite being aware of the physical and psychological harm caused by excessive exercise. EA is defined by the presence of three or more of the following seven criteria: (1) Tolerance; (2) Withdrawal symptoms; (3) Intention effects; (4) Lack of control; (5) Time spent exercising; (6) Reduction in other activities; (7) Continuance (Hausenblas and Downs, 2002).

Based on the criteria, the Exercise Dependence Scale (EDS) was developed to differentiate between exercise addiction risk and non-risk groups (Hausenblas and Symons Downs, 2002). The EDS was revised in 2004, can differentiate between risk, symptomatic, and asymptomatic groups. In addition, the psychometric properties of the scale were also improved (Downs et al., 2004). Exercise Dependence Scale-Revised (EDS-R) has been translated into various languages, including French, Portuguese, Spanish, Italian, among others (Allegre and Therme, 2008; Marques et al., 2019). It has been widely adopted and utilized globally, notably in countries such as Italy, Spain, and Turkey (Sicilia and González-Cutre, 2011; Costa et al., 2012; Orhan et al., 2019), demonstrating high validity and reliability. However, cross-cultural validation of the EDS-R remains relatively limited. Understanding and manifestations of exercise addiction may vary across different cultural backgrounds, necessitating further cross-cultural research to validate the effectiveness and adaptability of the EDS-R. Currently, research on the EDS-R has primarily focused on specific populations, such as athletes (McNamara and McCabe, 2012; Zimanyi et al., 2021). This limited sample may result in insufficient understanding of exercise addiction in other populations, such as university students, middle-aged and older adults, and non-competitive exercise participants. Epidemiological studies suggest that EA is not common in the general population, with a prevalence rate of 0.3–0.5%. However, research has found that EA is significantly more prevalent among university students and athletes, with prevalence rates of 3 to 9% (Landolfi, 2013). Research on the risk of exercise addiction in the Chinese population is scarce. A study conducted in the general population indicated a prevalence rate of 7.7% for exercise addiction (Yang et al., 2021), while an investigation focusing on Chinese university students revealed a significantly higher risk rate of 11.3% (Li, 2018). Therefore, further research is needed to explore the applicability and validity of the EDS-R in different populations. To confirm whether EDS-R can be used as a quantitative index for EA in the context of Chinese students, a survey was conducted among Chinese university students. The first objective was to test the reliability and validity of the Chinese version of EDS-R.

In light of the negative features of EA, a deeper understanding of the susceptibility variables associated with this disorder would be valuable. Knowledge of the factors related to EA can aid in better understanding the psychological mechanisms underlying this addiction and provide a basis for implementing prevention strategies. The motivation behind exercise has been specifically described as a key antecedent to EA (González-Cutre and Sicilia, 2012). Motivation determines the initiation, maintenance, and completion of relevant behaviors, and an analysis of motivation may be key to understanding EA (Conesa et al., 2017). Self-Determination Theory (SDT) is a foundational human motivation theory that is applicable for understanding both the quantity and quality of engagement in physical activities (Deci and Ryan, 2000). SDT introduces the concepts of intrinsic motivation and extrinsic motivation based on varying levels of motivational autonomy (González-Cutre and Sicilia, 2012). Intrinsic motivation refers to an individual’s engagement in an activity based on their own desires or personal interests, encompassing the inner drive that people exhibit when pursuing a pleasurable goal or activity. On the other hand, extrinsic motivation involves individuals engaging in a specific behavior driven by external forces, in pursuit of outcomes or rewards external to the activity itself (Teixeira et al., 2012).

Within this framework, Richard categorizes the motivations behind sports activities into five dimensions, with health and appearance falling under extrinsic motivation, while competence, social connections, and enjoyment fall under intrinsic motivation (Richard et al., 1997). This classification can assist us in better understanding the reasons that lead individuals to engage in sports and subsequently develop addictions. Research on the applicability of SDT in the realm of behavior indicates that addictive behavior in sports is positively correlated with intrinsic motivation, and it has become a significant predictor of sports participation (Granero-Jiménez et al., 2022; Sánchez-Herrera et al., 2022). Further research on the motivations for exercise that lead to exercise addiction, health deterioration, and a decrease in the quality of life is crucial. Therefore, the second objective of this study is to utilize the Multidimensional Physical Activity Motivation Scale-Revised (MPAM-R) within the framework of SDT theory to describe the impact of individual differences in motivation for exercise behavior on the risk of exercise addiction.

The purpose of this preliminary exploratory study is to examine the reliability and validity of the Chinese version of EDS-R among university students and to analyze their exercise frequency, exercise motives, and exercise addiction symptoms during leisure time. A study on different age groups of the Chinese population has shown that EDS-R can be an effective assessment tool with considerable practical value in screening EA (Yang et al., 2021). It is hypothesized that EDS-R is also applicable to university students in Chinese society and has good reliability and validity. Evidence from previous literature on exercise addiction suggests that males tend to exhibit more severe exercise addiction symptoms than females (Welch et al., 2007). SDT believes that intrinsic motivation is more likely to lead to primary EA, making individuals have a strong desire for motor activities and uncontrollable behavior (Wilson et al., 2003; Edmunds et al., 2006b); Whereas extrinsic motivation often leads to secondary EA, using movement as a surrogate for escape, control, or coping with other problems or dilemmas (Symons Downs et al., 2013). Therefore, it is hypothesized that male students would report higher exercise frequency, intrinsic motives, and exercise addiction symptoms than female students. Higher exercise frequency and motive scores may indicate a greater risk of EA.

## 2 Materials and methods

### 2.1 Participants

The participants of this study were native Chinese-speaking university students. Recruitment was conducted through poster advertisements on campus, email invitations sent via the school’s online platform, and oral promotion. Interested potential participants were provided with detailed research information and an informed consent form. The informed consent form explained the purpose, procedures, risks, and benefits of the study, and clarified the participants’ rights. Only those who understood and agreed to participate in the study were included. All enrolled participants completed the informed consent form, a demographic questionnaire, the Exercise Dependence Scale-Revised (EDS-R), and the Multidimensional Psychological Aspects of Physical Activity Motivation Scale-Revised (MPAM-R). Two independent researchers entered the participants’ data, cross-checked and verified the entries to identify and rectify any inconsistencies, ensuring the accuracy and consistency of the data. A total of 1,000 questionnaires were distributed for this experiment, among which 163 were deemed invalid due to incomplete filling, logical errors, language barriers, etc. These were excluded from the analysis. We successfully collected 837 valid questionnaires, resulting in an effective response rate of 83.70%.

### 2.2 Demographic data

Demographic items recorded participants’ gender, age, height, weight, and education level, Participants reported the number of times they participated in 30 min or more of physical activity per week, group by ≤3, >3 (Haskell et al., 2007). Body mass index (BMI), calculated from self-reported height and weight according to the Adolphe-Quetelet formula: weight (kg)/height (m)^2^.

### 2.3 Measurement tool

#### 2.3.1 The Exercise Dependence Scale-Revised (EDS-R)

The translation process involves several steps. Firstly, we engaged a professional translator whose native language is Chinese to translate the original English version of the scale. Consultations with sports psychologists were sought to ensure that the translated scale accurately conveys the meaning and intent of each item. Special attention was given to considering subtle cultural and linguistic differences to ensure the translation is appropriately understood in the target culture. Subsequently, a second translator, fluent in both English and Chinese as their native languages, conducted a back-translation of the Chinese version. The purpose of this step was to retranslate the Chinese version back into English, validating and ensuring that the translation preserves the core meaning and intent of the original scale. Emphasis was placed on maintaining consistency between the translated items and the original text. Following this, we formed an expert panel consisting of bilingual individuals and a psychologist to review both the original scale and the back-translated version. During the review process, the expert panel compared the two versions to identify any differences, inconsistencies, or subtle cultural nuances that may have arisen during the translation process. Any identified issues were discussed within the panel and resolved to ensure the accuracy and cultural appropriateness of the final translation. Through this systematic translation and review process, our aim is to maintain the precision of the scale across cultural contexts, enabling it to accurately convey the original scale’s meaning in the Chinese environment.

The EDS-R scale (Downs et al., 2004) was utilized to assess the subjects’ EA symptoms and risks. Each item on the scale was scored using the six-point Likert scale, ranging from one point for “never” to six points for “always” Participants were asked to rate their level of identification with each item based on their current sports beliefs and actions. In addition, the scale is divided into seven dimensions (withdrawal, continuance, tolerance, lack of control, reduction in activities, time, and intention effects) based on the fourth edition of the Diagnostic and Statistical Manual of Mental Disorders (DSM-IV) published by the American Psychological Association (Bell, 1994). Each of the three items measures a different dimension. If a dimension score is greater than 15 points, it indicates that the subject meets the symptom in its entirety; If the score is 7–14 points, it is partially consistent with the symptom; A score of 6 or less indicates that the subject does not meet the symptom’s criteria. If the number of symptoms is greater than or equal to three, then the subject is considered “at risk for exercise addiction.” If the number of fully compliant symptoms is less than three and the number of partially compliant symptoms is greater than or equal to three, the subjects are categorized as “non-addiction symptomatic,” while the remaining subjects are “non-addiction and asymptomatic.”

#### 2.3.2 The Motives for Physical Activity Measure-Revised Scale (MPAM-R)

The MPAM-R scale, revised by Ryan et al. in 1997, is a commonly used tool for measuring exercise motivation. Exercise motivation is the psychological motivation for people to engage in and maintain exercise behavior. Based on cognitive evaluation theory and the SDT, this scale provides a list of motivations for people to engage in physical exercise and physical activity (such as “I want to improve my physical fitness”) and allows subjects to evaluate the strength of these motivations. The MPAM-R consists of 15 items covering five dimensions: health, appearance, ability, social, and enjoyment. On a scale ranging from 1 (strongly disagree) to 5 (strongly agree), participants’ agreement with the motivation was measured according to the 5-point Likert scale. Each dimension’s score is derived by summing the scores of its constituent items. The higher the score, the greater the level of exercise motivation that this dimension represents. Existing studies have demonstrated the reliability of the Chinese version of the MPAM-R scale (Chen et al., 2013). In this study, the overall reliability of McDonald’s ω of the scale was 0.948.

### 2.4 Data analysis

After entering the original data into an Excel spreadsheet, SPSS (Statistical Package for Social Science (SPSS), Version 26, Chicago, Illinois, United States) was used for data analysis. First, descriptive statistics (mean, standard deviation, kurtosis, and skewness) for each item and dimension of EDS-R were calculated. Second, reliability and validity tests were conducted on the EDS-R, including McDonald’s ω , which represents internal consistency (Roco-Videla et al., 2023), as well as reliability tests on the MPAM-R scales. Using the Amos 23.0 plug-in, a confirmatory factor analysis of the Chinese version of EDS-R was conducted to calculate the load factors for each item, combined reliability (CR), and average extraction variance (AVE) of each dimension and total scale (Conway, 2020). The maximum likelihood method was utilized to compute the model’s goodness-of-fit statistics and goodness-of-fit index: chi-square value (χ2), the absolute fitting index (χ2/df), the root means the square error of approximation (RMSEA), the non-normed fitting index (NNFI), the comparative fitting index (CFI), and the incremental fitting index (IFI) (Alavi et al., 2020).

The Kruskal-Wallis H test was used to compare the differences in MPAM-R scores between groups, and the Bonferroni method was used to correct the significance level. The partial correlation coefficient was employed to assess the relationship between Chinese version of EDS-R score and exercise frequency, and exercise motivation. Performing the Mann–Whitney U test between the male and female groups to determine the underlying cause of the gender gap in EA. Verify the predictive effect of exercise frequency, and exercise motivation on exercise dependence based on gender, age, and BMI using hierarchical regression analysis.

### 2.5 Ethics

This study was conducted in strict accordance with the Declaration of Helsinki and approved by the Ethics Committee of the First Hospital of Shanxi Medical University. Informed consent has been signed by all subjects.

## 3 Results

### 3.1 Descriptive statistics of the participants

This study included 837 college students, 281 of whom were male (33.6%), and 556 of whom were female (66.4%), the average age was (20.38 ± 2.36) years. The basic characteristics of the study participants, including gender, age, BMI, and frequency of weekly exercise (Table 1). In this study, the EDS classification identified 41 people (4.9%) with risk and symptomatic exercise addiction. Among them, 17 were male and 24 were female (In males, the incidence is 6.0%, In females, it’s 4.3%). 356 people (42.5%) with no risk and symptoms, and 440 with no risk and asymptomatic exercise addiction (52.6%).

**Table 1:** Descriptive statistics of the participants.

	Participants	Quantity	Percentage (%)
Age	≤18	131	15.7
	19–22	618	73.8
	23–26	71	8.5
	≥27	17	2.0
Gender	Male	281	33.6
	Female	556	66.4
BMI	<18.50	183	21.9
	18.50–23.99	535	63.9
	24.00–27.00	83	9.9
	>27.00	36	4.3
Exercise frequency	≤3	631	75.4
	>3	206	24.61

### 3.2 Reliability and validity test of EDS-R scale

#### 3.2.1 Descriptive statistics and reliability analysis of Chinese version of EDS- R scale

As shown in Tables 2, 3, descriptive statistics were calculated for each scale item and dimension score. The fact that the mean value of each item on the scale ranges from 1.80 to 3.19, the standard deviation ranges from 1.23 to 1.45, and the kurtosis and skewness of each item are between −2 and 2, demonstrates that EDS- R scores conform to a normal distribution (Potenza et al., 2013). McDonald’s ω was calculated for each dimension and the total scale. The McDonald’s ω for each dimension ranged from 0.87 to 0.97, and for the total scale was 0.973. According to the load factors of each item, CR and AVE of each dimension and the total scale were calculated. The CR of each dimension ranged from 0.87 to 0.96, while the AVE ranged from 0.69 to 0.90, with a total value of CR = 0.99 and AVE = 0.80., the greater the reliability of the scale. If the McDonald’s ω is below 0.7, it is considered insufficient for internal consistency and deemed unacceptable (Roco-Videla et al., 2023). In this study, the reliability is between 0.87 and 0.97, it is acceptable (Peters, 2014). CR > 0.7 and AVE > 0.5 are indicative of the scale’s high reliability (Fornell and Larcker, 1981), all scale dimensions and total scale indicators meet the standards. It can be concluded that all scale dimensions and the total scale are reliable.

**Table 2:** Descriptive analysis and normality test of EDS-R.

	M(SD)	Ske	Kur	FL
Withdrawal				
1. I exercise to avoid irritable	2.51(1.45)	0.704	−0.43	0.722
8. I exercise to avoid feeling anxious	1.99(1.31)	1.25	0.73	0.902
15. I exercise to avoid feeling tense	2.25(1.38)	1.04	0.29	0.840
Continuance				
2. I exercise despite recurring physical problem	2.04(1.30)	1.27	0.92	0.834
9. I exercise when injured	2.40(1.40)	0.80	−0.22	0.806
16. I exercise despite persistent physical problems	2.66(1.45)	0.57	−0.57	0.447
Tolerance				
3. I continually increase my exercise intensity to achieve the desired effects/benefits	3.13(1.40)	0.26	−0.72	0.804
10. I continually increase my exercise frequency to achieve the desired effects/ benefits	1.96(1.29)	1.35	1.13	0.865
17. I continually increase my exercise duration to achieve the desired effects/ benefits	3.19(1.39)	0.21	−0.71	0.767
Lack of control				
4. I am unable to reduce how long I exercise	1.91(1.25)	1.42	1.34	0.892
11. I am unable to reduce how often I exercise	3.16(1.37)	0.23	−0.68	0.886
18. I am unable to reduce how intensely I exercise	1.91(1.26)	1.50	1.66	0.920
Reduction in activities				
5. I would rather exercise than spend time with family/friends	1.96(1.29)	1.34	1.12	0.704
12. I think about exercise when I should be concentrating on school/work	1.92(1.23)	1.43	1.51	0.707
19. I choose to exercise so that I can get out of spending time with family/friends	2.13(1.31)	1.06	0.33	0.741
Time				
6. I spend a lot of time exercising	2.09(1.33)	1.11	0.37	0.818
13. I spend most of my free time exercising	2.11(1.30)	1.11	0.50	0.881
20. A great deal of my time is spent exercising	1.80(1.25)	1.66	1.98	0.827
Intention effects				
7. I exercise longer than I intend	1.99(1.31)	1.25	0.73	0.823
14. I exercise longer than I expect	2.08(1.30)	1.17	0.64	0.845
21. I exercise longer than I plan	2.14(1.32)	1.11	0.51	0.845
Total	47.53(22.37)	1.24	1.50	

*N* = 837 for all the items. M, Mean; SD, Standard Deviation; Ske, Skewness; Kur, Kurtosis; FL, Factor Loadings.

**Table 3:** Normality analysis and reliability and validity test of seven dimensions in EDS-R.

Subscales	M(SD)	Ske	Kur	ω	CR	AVE
Withdrawal	7.16(3.96)	0.84	0.01	0.867	0.93	0.82
Continuance	6.65(3.55)	1.11	0.82	0.941	0.87	0.69
Tolerance	9.47(3.89)	0.22	−0.48	0.931	0.93	0.82
Lack of control	5.75(3.62)	1.45	1.65	0.965	0.96	0.90
Reduction in activities	6.20(3.52)	1.18	0.95	0.939	0.88	0.72
Time	5.97(3.67)	1.36	1.24	0.885	0.94	0.84
Intention effects	6.33(3.79)	1.04	0.36	0.929	0.94	0.84
Total	47.53(22.37)	1.24	1.50	0.972	0.99	0.80

*N* = 837 for all the items. M, Mean; SD, Standard Deviation; Ske, Skewness; Kur, Kurtosis; ω, McDonald’s ω; CR, combined reliability; AVE, average extraction variance.

#### 3.2.2 Confirmatory factor analysis of the EDS-R scale

**Figure 1:**
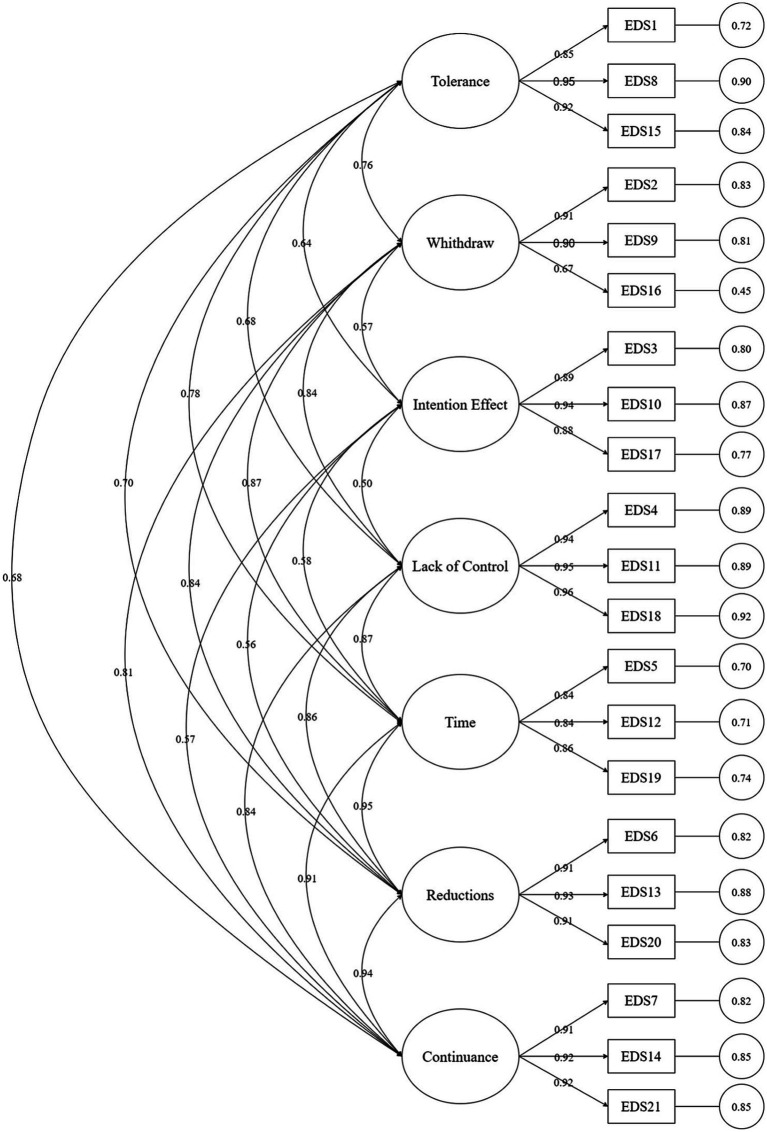
The Confirmatory factor analysis model of Chinese version of EDS-R. The structure of the validity diagram consists of ellipses representing seven dimensions and bidirectional arrows indicating the correlation between these dimensions. The numbers on the arrows reflect the correlation coefficients between the seven dimensions. The squares represent the measurement items, and the numbers on the unidirectional arrows indicate the path coefficients; The values inside the circles denote the factor loadings.

The maximum likelihood method (Dempster et al., 1977) was used to calculate the goodness-of-fit statistics and goodness-of-fit index of the model, and the following values were obtained: χ^2^ = 1050.763, χ^2^/df = 5.773, RMSEA = 0.076, NNFI = 0.950, CFI = 0.958, IFI = 0.968. Among these, the χ^2^ value is greatly influenced by sample size and is generally considered acceptable if χ^2^/df is less than 8 (Alavi et al., 2020). It has been suggested that the RMSEA value should be less than 0.08, with smaller values indicating a better fit between the model and the data. The NNFI, CFI, and IFI indices also typically range from 0 to 1, with values closer to 1 indicating a better fit between the model and the data, generally above 0.90 (Hu and Bentler, 1999; McDonald and Ho, 2002). According to the comparison with the suggested value, the model corresponds well with the data. The items of the scale are unambiguous, and the load factors are all greater than 0.6 (Fu et al., 2022) according to confirmatory factor analysis (Table 2). All loading factors were statistically significant at the *p* = 0.01 level, indicating that the scale has good structural validity. Figure 1 illustrates the structure of confirmatory factor analysis.

### 3.3 Analysis of psycho-social factors related to EA

Based on the EDS-R scoring criteria, participants were categorized into three groups: “1 = symptomatic and at risk, 2 = symptomatic but not at risk, 3 = asymptomatic and not at risk.” The Kruskal-Wallis H test was employed to examine differences in MPAM-R scores among the groups (Table 4). The findings revealed significant differences among the three groups in various aspects of exercise motivation, such as appearance (*H* = 7.850, *p* = 0.020), pleasure (*H* = 12.911, *p* = 0.002), ability (*H* = 45.615, *p* < 0.001), and social factors (*H* = 10.350, *p* = 0.006), while differences in health (*H* = 4.941, *p* = 0.085) were not statistically significant. Using the Bonferroni method to adjust for multiple comparisons, the significance level of post-hoc test was reduced, revealing significant differences only in ability among the three groups (*p* < 0.001).

**Table 4:** A group comparison between negative emotion and motor motivation.

	Kruskal- Wallis H		Bonferroni (adj.p)		
	*H*	*p*	1–2	1–3	2–3
Healthy	4.914	0.085	–	–	–
Appearance	7.850	0.020	0.021	0.100	0.511
Pleasure	12.911	0.002	0.002	<0.001	1.000
Ability	45.615	<0.001	<0.001	<0.001	<0.001
Social	10.350	0.006	0.103	0.010	0.193

Given that EA is significantly linked to gender, with males having a greater susceptibility to EA than females, we divided the participants into male and female groups based on their gender and used the Mann–Whitney U test to investigate possible factors. The results revealed that men exhibited higher exercise frequency (*p* < 0.001) and stronger healthy motivation (*p* = 0.044) and ability motivation (*p* = 0.025) than women, while women demonstrated greater appearance motivation (*p* < 0.001; Table 5).

**Table 5:** Comparison of differences in risk factors between male and female group.

	Male (*N* = 281)	Female (*N* = 556)	*z*	*p*
	Mean rank	Mean rank		
Frequency	468.50	393.98	−4.498	<0.001^*^
Healthy	442.00	407.38	−2.015	0.044^*^
Appearance	378.32	439.56	−3.549	<0.001^*^
Pleasure	433.55	411.65	−1.267	0.205
Ability	444.90	405.91	−2.242	0.025^*^
Social	430.78	413.04	−1.029	0.303

*p < 0.05.

### 3.4 Hierarchical regression model for predicting EDS- R

In addition, hierarchical regression analysis confirmed the relationship between EA, exercise frequency, and exercise motivation, which was able to predict 15.5% of the variance in exercise addiction scores. The first block incorporated demographic variables, including gender, age, and BMI, which accounted for 4.9% of the variance in EDS-R scores. The second block included exercise frequency, which explained an additional 5.6% of the variance related to EA (*β* = 0.240, Δ*R*^2^ = 0.056, *p* < 0.001). In the third block, exercise motivation was added to the block on top of exercise frequency, explaining an additional 5.0% of the variance in exercise addiction. Furthermore, ability motivation demonstrated a significant positive correlation with exercise addiction scores (*β* = 0.336, *p* < 0.001), which is consistent with the results of the Kruskal-Wallis H test (Table 6).

**Table 6:** Hierarchical regression model to predict EDS-R.

		*β*	*t*	Δ*R*^2^	*R* ^2^	*F*
Block 1				—	0.049	14.293^***^
	BMI	0.044	1.265			
	Gender	−0.099	−2.854^**^			
	Age	0.176	5.170^***^			
Block 2				0.056	0.105	24.292^***^
	Frequency	0.240	7.189^***^			
Block 3				0.050	0.155	16.893^***^
	Healthy	−0.110	−1.892			
	Appearance	−0.056	−1.022			
	Pleasure	−0.047	−0.648			
	Ability	0.336	6.491^***^			
	Social	−0.023	−0.355			

**p < 0.01, ***p < 0.001. BMI, body mass index.

## 4 Discussion

Our study investigated the reliability and validity of the Chinese version of the EDS-R scale for assessing exercise addiction risk among Chinese university students and analyzed the relationship between exercise frequency, exercise motivation, and EA. Our findings suggest that the Chinese version of EDS-R scale is a reliable and valid tool for assessing exercise addiction risk among university students and provides a valuable tool for future research and clinical practice. Additionally, our results revealed gender differences in exercise addiction prevalence, with men exhibiting higher risk than women. This highlights the importance of gender as a potential moderating factor in the development of EA. Further analysis suggests that exercise motives may play a critical role in understanding these gender differences, as men scored higher than women on ability motivation. Overall, our study contributes to a better understanding of EA and its underlying.

This study examined the reliability and validity of the Chinese version of the EDS-R in a sample of Chinese university students. Using McDonald’s ω coefficient, we evaluated the internal consistency of the total scale and each subscale and found that both the total scale and each subscale exhibited high reliability. We also conducted a confirmatory factor analysis to assess the seven dimensions of the scale. The goodness of fit of the model was evaluated by calculating the maximum likelihood estimates of the factor loadings, the CR and AVE as reliability indices, and the CFI and RMSEA as structural validity indices. Our results indicated that the scale had acceptable reliability and structural validity indices and replicated the same factor structure as the original EDS-R version. The prevalence of EA risk in our study was 4.9%, align with the results of a systematic review conducted by Marques et al. They comprehensively analyzed relevant literature pertaining to European countries and reported a prevalence range of 3–7% for exercise addiction among university students (Marques et al., 2019). The sustained prevalence of EA in several countries suggests that it may be a cross-cultural phenomenon, and the development of psychometrically sound versions of the EDS-R in other languages may deepen our understanding of this phenomenon from a cross-national perspective. In summary, the Chinese version of the EDS-R is a reliable and valid measurement tool that can be used to assess exercise addiction symptoms in Chinese populations.

The results of a hierarchical regression model suggest that exercise frequency and exercise motivation both contribute to the development of EA. Our findings indicate that exercise frequency accounts for 5.6% of EA, indicating that high exercise frequency is a significant factor in EA. In exercise addiction, high-frequency exercise can lead to neuroadaptation and changes in neurotransmitters (Szabo and Demetrovics, 2022). Specifically, individuals with EA produce large amounts of endogenous opioid-like substances such as endorphins and enkephalins during exercise, which can bind to neurotransmitters and influence the brain’s activity in regions associated with EA. Long-term high-frequency exercise may lead to neuroadaptation, requiring higher exercise intensity to achieve the same level of endogenous opioid-like substance release, further promoting the development of EA (Ding et al., 2019). Additionally, research indicates that individuals with EA have elevated levels of dopamine, a neurotransmitter associated with reward and motivation (Weinstein and Weinstein, 2014). High-frequency exercise may cause excessive stimulation of dopamine receptors, leading to an increased exercise frequency to achieve the same level of dopamine release, which may also promote the development of EA. Compared to exercise frequency, the impact of exercise duration on EA may be less significant. Although prolonged exercise can increase reward response and endogenous opioid-like substance secretion, a study found that short-term exercise can also elicit reward response, triggering cravings and dependency on exercise, leading to the onset of EA (Duncan et al., 2010; Bueno-Antequera et al., 2020).

Exercise motivation is an essential factor in promoting and sustaining physical activity, this study suggests that the influence of exercise motivation on EA is mainly reflected in the areas of ability motivation. In the context of sports, ability motivation refers to an individual’s drive to improve and develop their athletic abilities. This internal drive is fueled by their focus and interest in their performance level and their intrinsic motivation to enhance their abilities (Staples et al., 2022). Research has found that individuals with EA exhibit high levels of self-challenge and competitiveness, which are associated with ability motivation. This motivation may lead to a continuous pursuit of higher athletic achievement and physical performance, thereby facilitating the development of addiction behavior (Edmunds et al., 2006a). Moreover, individuals with EA often display excessive self-expectations and self-evaluation, consistent with the expression of ability motivation (Edmunds et al., 2006a). Additionally, ability motivation can foster personal growth and development, enhancing self-esteem and self-confidence, and strengthening an individual’s adaptability and coping ability. However, lacking self-confidence and coping ability in other areas of life may lead individuals to utilize sports as a means of fulfilling their needs (Li, 2018). The sense of achievement and self-worth that individuals derive from their performance in sports is closely related to their ability motivation and athletic performance. Negative experiences, such as setbacks or failures, can result in feelings of frustration and self-doubt, increasing their reliance on exercise and the potential risk of addiction.

In addition, our study confirmed the previous hypothesis that while both males and females may be affected by exercise addiction, the incidence rate of EA in males is significantly higher than in females. The reasons for this gender difference may be related to biological, social, and psychological factors. The male body structure is more suitable for high-intensity exercise, such as endurance and strength training, furthermore, males may be more inclined to seek adventure and excitement in exercise, which may increase their risk of developing EA. Males may be more likely to be pressured by coaches, peers, and family members in society (Meulemans et al., 2014) to maintain a strong physique and a high level of competitiveness. Studies have shown that (Hausenblas et al., 2017) there are also some differences between males and females in exercise motivation and emotional experience, which are also one of the reasons for gender differences in EA. Males usually have higher ability motivation, which is the pursuit of challenge, competition, and a sense of achievement, while females tend to have more appearance motivation, which is for body shape, appearance, and health reasons. This difference in motivation makes males more likely to develop primary exercise addiction, which is out of their interest and needs, while females are more likely to be stimulated by external factors, such as social pressure, media promotion, and social environment, to develop secondary exercise addiction (Cook et al., 2014). Therefore, gender differences play an important role in the occurrence and development of EA and should be considered in the prevention and treatment of EA.

However, there are still some issues that need to be further investigated. Firstly, the present study used self-report measures, which may be subject to recall bias and social desirability bias and may be influenced by individual subjective feelings. Secondly, the study did not take into account the influence of cultural background and exercise experience on EA, which needs to be further explored in future research. Thirdly, because there were few types of exercise, we did not differentiate between exercise types, although previous research has shown that exercise types also affect the incidence of EA (de la Vega et al., 2016). In the future, we will expand the inclusion of individuals with different exercise types to investigate their correlations. Fourth, the proportion of females in the sample for this study is relatively high. In future research, we plan to increase the inclusion of male participants to achieve a more balanced gender distribution. Additionally, we will conduct further confirmatory factor analysis to assess various forms of invariance. Fifthly, this study was a cross-sectional study and cannot reveal the long-term process and dynamic changes of EA development, and longitudinal studies are needed to explore the changes in the developmental trajectory of EA influenced by different factors.

## 5 Conclusion

In summary, our study asserts that the Chinese version of the EDS-R is a reliable tool for screening individuals at risk of EA in the Chinese context. High exercise frequency and ability motivation are identified as risk factors for the occurrence of EA, findings that hold the potential to enhance societal identification of individuals at risk of EA. Furthermore, our research unveils gender differences in exercise motivation. Considering the impact of gender disparities on the risk of EA, future intervention strategies could be tailored to address the unique needs of different genders. Our study holds practical significance in guiding the development of more effective interventions and preventive measures, providing substantial support for the promotion of healthy lifestyle choices.

## Data availability statement

The raw data supporting the conclusions of this article will be made available by the authors, without undue reservation.

## Ethics statement

The studies involving humans were approved by the Ethics Committee of the First Hospital of Shanxi Medical University. The studies were conducted in accordance with the local legislation and institutional requirements. The participants provided their written informed consent to participate in this study.

## Author contributions

YS: Investigation, Software, Writing – original draft. HaZ: Data curation, Formal Analysis, Writing – original draft. XZ: Data curation, Methodology, Writing – original draft. QL: Writing – original draft. FZ: Writing – review & editing. HuZ: Project administration, Validation, Writing – review & editing.
